# Group A Streptococcal Infections in Pediatric Age: Updates about a Re-Emerging Pathogen

**DOI:** 10.3390/pathogens13050350

**Published:** 2024-04-24

**Authors:** Giada Maria Di Pietro, Paola Marchisio, Pietro Bosi, Massimo Luca Castellazzi, Paul Lemieux

**Affiliations:** 1 Pediatric Unit, Fondazione IRCCS Cà Granda Ospedale Maggiore Policlinico, 20122 Milan, Italy; 2 Department of Clinical Sciences and Community Health, Università degli Studi di Milano, 20122 Milan, Italypaul.lemieux@unimi.it (P.L.); 3 Pediatric Emergency Department, Fondazione IRCCS Cà Granda Ospedale Maggiore Policlinico, 20122 Milan, Italy; luca.castellazzi@policlinico.mi.it

**Keywords:** *Streptococcus pyogenes*, group A *Streptococcus* (GAS), pharyngitis, invasive group A streptococcal infection (iGAS), vaccines

## Abstract

Group A Streptococcus (GAS) presents a significant global health burden due to its diverse clinical manifestations ranging from mild infections to life-threatening invasive diseases. While historically stable, the incidence of GAS infections declined during the COVID-19 pandemic but resurged following the relaxation of preventive measures. Despite general responsiveness to β-lactam antibiotics, there remains an urgent need for a GAS vaccine due to its substantial global disease burden, particularly in low-resource settings. Vaccine development faces numerous challenges, including the extensive strain diversity, the lack of suitable animal models for testing, potential autoimmune complications, and the need for global distribution, while addressing socioeconomic disparities in vaccine access. Several vaccine candidates are in various stages of development, offering hope for effective prevention strategies in the future.

## 1. Epidemiology

*Streptococcus pyogenes* (also known as Group A Streptococcus (GAS)) is a major human-specific Gram-positive coccus responsible for a wide range of diseases differing in clinical presentation and severity [1]. GAS can colonize the skin and throat, causing asymptomatic and self-limited conditions or symptomatic infections. The latter can vary from superficial infections of the throat (pharyngitis and tonsillitis) and skin (impetigo, pyoderma, and cellulitis) to serious life-threatening invasive infections, collectively termed invasive group A streptococcal (iGAS) diseases and defined as severe illnesses associated with the isolation of GAS from a normally sterile site, such as blood, cerebrospinal fluid, deep muscle, or pleural fluid (causing necrotizing fasciitis, osteomyelitis, and bacteremia) [2,3]. GAS infections can also cause autoimmune sequelae after the production of specific antibodies, such as acute rheumatic fever (ARF) and rheumatic heart disease (RHD), both of which are related to antibody-directed molecular mimicry with abnormal host immune response, as well as post-streptococcal glomerulonephritis (APSGN) consequent to the deposition of immune complexes [4].

The variety of the clinical manifestations may originate from the large strain diversity, as more than 250 *emm*-types have been described. The highest genetic diversity of circulating strains has been observed in developing countries, while Europe and North America show the lowest strain heterogeneity. Recent studies conducted in Europe and Australia in 2022 and 2023, respectively, identified *emm*-type 1 and *emm*-type 12 as more associated with invasive disease, while a systematic review of studies on bacterial samples originating from 55 countries and collected from 1990 to 2023 demonstrated that *emm*-type 1 ranked 39th among all *emm*-types associated with invasive disease and identified other *emm*-types as emergent causes of iGAS in the world. The same authors recognized 15 *emm*-clusters that accounted for 95.6% of the strain diversity in the global, population-weighted, *emm*-type dataset and concluded that the development of a vaccine protective against them would theoretically protect against more than 95% of the disease-causing strains, underlining the importance of knowledge of the *emm*-type distribution worldwide [5,6,7,8,9].

GAS is the principal bacterial cause of tonsillopharyngitis in children all over the world: in 2005, the global burden of *Streptococcus pyogenes* sore throat among children aged 5–14 years was 446 million episodes each year, which decreased to 288.6 million in 2022 [10]. Usually, the incidence of GAS pharyngitis peaks during winter and in the first part of spring [11]. GAS tonsillitis predominantly affects school-age children, though younger children, especially those in contact with school-age children, are also susceptible to infection [12]. Prompt antibiotic treatment of GAS in children, after microbiologic confirmation, is necessary to prevent complications, further disease transmission, and deaths. Invasive GAS infections can rapidly worsen if left untreated, often necessitating surgical intervention to be fully controlled [13]. A recent multicenter cohort study including 320 children with iGAS infections reported an overall mortality rate of 2%, with 12% experiencing survival with neurodisability, amputation, skin grafts, hearing loss, and the need for surgery [14].

The annual burden of iGAS disease and RHD is 663,000 and 282,000 new cases worldwide, respectively. Based on data for the 2010s, each year, Group A *Streptococcus* is responsible for 163,000 deaths due to iGAS and 345,000 deaths due to RHD, making it the fifth most lethal pathogen in the world [2]. While the incidence of GAS infections has historically remained stable, there was a decline during the pandemic period. However, following the relaxation of preventive measures against COVID-19, a significant resurgence of GAS infections was observed worldwide. In late 2022, many countries reported a considerable increase in scarlet fever and iGAS infections, particularly affecting children under 10 years of age, raising concerns about the high iGAS incidence and mortality among children [15,16]. Between late 2022 and early 2023, an increased incidence of iGAS infections was also reported in the Milan area [17].

Two years of social distancing and barrier measures needed to fight the COVID-19 pandemic caused a decrease in exposure to pathogens, including GAS [18]. This reduction may have resulted in a state of reduced immunity, termed “immune debt”, potentially contributing to increased iGAS infection incidence among susceptible children [19].

The upsurge in incidence appears unrelated to any predominant GAS strain but rather correlates with this “immune debt” following reduced exposure to both GAS and common viral respiratory infections [20]. The recrudescence of predisposing viral infections after COVID-19 has increased the prevalence of iGAS infections; most reported cases are superinfections of viral respiratory infections [18,21]. Recent studies showed that a coexistent viral respiratory tract infection was present in up to 60% of iGAS cases in children [22]. A link with preceding or coinciding varicella was highlighted in a recent Dutch study [5].

**Figure 1:**
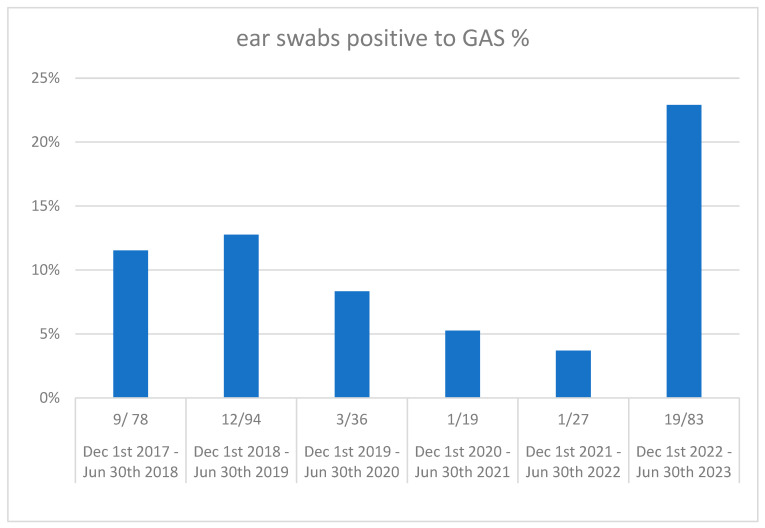
Ear swabs positive for GAS in a tertiary care hospital in northern Italy in five consecutive seasons (2018–2023).

Supporting this trend, an increased number of cases of acute otitis media (AOM) with otorrhea have been noted in children regularly followed for recurrent AOM at our tertiary outpatient clinic for upper respiratory tract infections since late 2022 [23]. We retrospectively analyzed ear swab results obtained for otorrhea in our Pediatric Emergency Room and outpatient clinics from 1 December 2022 to 30 June 2023. We considered children from 1 month to 6 years of age due to the predominant prevalence of AOM in the first years of life [24]. Comparison with results from the same months of previous years revealed a significant rise in the prevalence of GAS-positive ear swabs during the 2022–2023 season (Figure 1).

Using the chi-squared test, we also compared the proportion of GAS-positive ear swabs during the 2022–2023 season with the overall proportion for the previous five seasons (GAS-positive swabs in the five seasons divided by total swabs in the same period), which revealed a significant percent increase in the 2022–2023 season (23% vs. 10%, *p* < 0.01).

Corroborating previously published findings, we also noticed a significant drop in AOM with otorrhea cases during the COVID-19 pandemic as a result of the global improvement of otitis-prone children in Milan during lockdown [25]. Additionally, the prevalence of *Streptococcus pyogenes*-positive ear swabs during the pandemic significantly decreased, likely as a result of reduced exposure of children to GAS.

## 2. Clinical Features

GAS causes a wide range of clinical infections ranging from mild to severe and potentially fatal, affecting various organ systems and encompassing superficial, invasive, and toxin-mediated diseases. The clinical manifestations of GAS infection are discussed in the following subsections and broken down by clinical diagnosis [26].

## 3. Non-Invasive Group A Streptococcal Infections

### 3.1. Streptococcus pyogenes Pharyngitis

*Strepyococcus pyogenes* throat is a prevalent respiratory infection, predominantly affecting children aged 5 to 15 years. Streptococcal pharyngitis typically presents with an abrupt onset of symptoms, including pharyngodynia, malaise, fever, and headache. In children under 5, common symptoms also include abdominal pain, nausea, and vomiting. Cough, coryza, and conjunctivitis are atypical symptoms of streptococcal pharyngitis and are more often indicative of a viral etiology [27]. The use of clinical scores, such as the Centor score, in aiding diagnosis remains controversial due to their limited sensitivity and specificity. The Centor criteria include four clinical parameters, each scoring one point: tonsillar exudate, tender anterior cervical lymphadenopathy or lymphadenitis, fever (over 38 °C), and absence of cough. The modified Centor criteria, also called the Mc Isaac score, include age as an additional criterion and are a clinical aid for physicians to determine who to test and treat when GAS pharyngitis is suspected [28]. Pharyngodynia in streptococcal pharyngitis is often asymmetric and may be severe. The presence of intense unilateral pain or difficulty swallowing, especially more than 3–5 days after the onset of fever, should prompt consideration of a local suppurative complication, such as a peritonsillar or retropharyngeal abscess [29].

In children under 3 years of age, streptococcal pharyngitis rarely presents with exudative pharyngitis. Instead, manifestations may include coryza, excoriated nares, and generalized adenopathy. While specific treatment can expedite recovery, in most cases, fever resolves within 3 to 5 days, and throat pain typically improves within a week, even without specific intervention [27].

### 3.2. Scarlet Fever

Scarlet fever is most common in children 5 to 15 years of age and is found rarely in children under 3. A disease caused by erythrogenic toxins produced by GAS, scarlet fever manifests as a finely papular (1 to 2 mm), blanching erythematous rash that gives a “sandpaper” texture to the skin [30]. The rash generally spares the face, palms, and soles and is often more pronounced in intertriginous areas, followed by a period of desquamation during recovery. In the antecubital fossa and axillary folds, the rash frequently has a linear, petechial quality, known as Pastia’s lines [30]. Circumoral pallor and oral mucositis in the form of a white or, at later stages, red strawberry tongue are frequent findings. Scarlet fever is often associated with streptococcal pharyngitis. Due to significant similarity in symptoms and age ranges affected, SARS-CoV-2 multisystem inflammatory syndrome (MIS-C) should be included within the differential diagnosis.

### 3.3. Rheumatic Fever

ARF is an inflammatory condition that arises as a consequence of inadequately treated or untreated GAS infections, particularly pharyngitis. The disease primarily targets the connective tissues of the heart, joints, skin, and subcutaneous tissues [26]. The hallmark of ARF is its propensity to involve the heart, resulting in rheumatic carditis. Patients may present with a variety of cardiac symptoms, ranging from mild to severe. Common cardiac manifestations include pancarditis (involvement of the pericardium, epicardium, myocardium, and endocardium) and valvulitis, particularly affecting the mitral and aortic valves. Cardiac involvement is generally apparent within 3 weeks of GAS infection [31]. Rheumatic fever can also affect large joints such as the knees, ankles, elbows, and wrists. Typically migratory in nature, arthritis may shift from one joint to another over a short period. Pain, swelling, and increased temperature are typical, and while the symptoms are usually self-limiting, their recurrence can contribute to chronic joint damage [2]. Dermatological manifestations of ARF include erythema marginatum and subcutaneous nodules. Erythema marginatum presents as pink, non-pruritic, serpiginous lesions with a clear center and well-demarcated borders, typically affecting the trunk and proximal extremities. Subcutaneous nodules, on the other hand, are firm, painless, and may be found over extensor surfaces and joints. Lastly, acute rheumatic fever can result in Sydenham’s chorea, characterized by rapid, uncoordinated movements [31]. Diagnosis is aided by use the of the modified Jones criteria, demonstrating improved accuracy in clinical diagnosis [32].

### 3.4. Post-Streptococcal Glomerulonephritis

APSGN is an immune-mediated inflammatory response affecting the glomeruli of the kidneys, typically occurring after an episode of pharyngitis or impetigo caused by nephritogenic strains of GAS. The onset of APSGN generally occurs 1 to 3 weeks following a streptococcal infection. The hallmark of APSGN is its impact on renal function, characterized by hematuria, proteinuria, and edema. Hematuria is often the initial presenting symptom. Proteinuria is typically mild to moderate, and edema may manifest as localized facial edema or generalized swelling, reflecting the underlying renal pathology. Hypertension is a common accompaniment to APSGN and may contribute to the progression of renal damage. APSGN is characterized histologically by diffuse proliferative glomerulonephritis with hypercellularity, “lumpy–bumpy” immunofluorescence deposits, and subepithelial humps on electron microscopy, indicative of immune complex deposition involving both streptococcal antigens and components of the glomerular basement membrane [26,33].

### 3.5. Impetigo

Impetigo is a common skin infection in children ages 2 to 5 years old. Non-bullous impetigo is more frequently caused by *Staphylococcus aureus* (80% of cases), but in a minority of cases (10%) it is caused by *Streptococcus pyogenes* [34]. The initial lesion is a vesicle on an erythematous base, easily ruptured, leading to superficial ulceration covered by purulent discharge that forms an adhering, honey-colored crust, most commonly on the face and extremities. Lesions typically measure 1 to 2 cm in diameter, growing centrifugally. Satellite lesions caused by self-inoculation are common. Impetigo may be a primary infection of the skin or may secondarily infect atopic dermatitis, contact dermatitis insect bites, pediculosis, herpetic lesions, or scabies. Regional lymphadenopathy is frequent, and severe cases may exhibit fever. Malnutrition and poor hygiene are predisposing factors [35]. Non-bullous impetigo typically resolves within two to three weeks without scarring.

### 3.6. Cellulitis and Erysipelas

Cellulitis is an infection of the dermis and subcutaneous tissues, presenting as localized inflammation, characterized by warmth, erythema, pain, and lymphangitis. The disease can progress to systemic involvement, marked by fever and an elevated white blood cell count. Erysipelas, categorized as a type of cellulitis, is distinguished by pronounced superficial inflammation, with the term commonly employed when facial involvement is noted. While cellulitis and erysipelas predominantly target the lower limbs, the ears, trunk, fingers, and toes are also involved to a less frequent extent [36].

## 4. Invasive Group A Streptococcal Infections

Although GAS infections in the great majority of cases are localized infections of the oropharynx or soft tissues, invasive systemic infections from GAS occur via direct extension into a normally sterile site, such as blood, cerebrospinal fluid, deep muscle, or pleural fluid, as is the case of necrotizing fasciitis, streptococcal bacteremia, and the endotoxin-mediated, fulminant Streptococcal toxic shock syndrome (STSS) [3]. Other forms of iGAS infections include GAS meningitis and septic arthritis.

### 4.1. Necrotizing fasciitis

Necrotizing fasciitis, an infrequent soft-tissue infection, manifests as swift inflammation leading to necrosis of the epidermis, dermis, subcutaneous tissue, and muscle fascia. The course of necrotizing fasciitis typically adheres to a defined sequence. Initial signs and symptoms include diffuse erythema and swelling, accompanied by intense tenderness and pain. Subsequently, clear, fluid-filled bullae emerge, which turn maroon or violet in color. This progression is succeeded by cutaneous gangrene, evolving rapidly alongside an extension of inflammation. Lymphangitis and lymphadenitis are infrequent. Necrotizing infection typically presents acutely with progression over hours. Rarely, cases of subacute progression over days have been documented [37]. Necrotizing infection most commonly involves the extremities. Whether the upper or lower extremities are more commonly involved is unclear, as observations vary between studies, and this aspect may change over time [38,39]. Risk factors include diabetes and/or peripheral vascular disease. Rapid progression can occur, leading to systemic involvement, limb loss, or death. While often triggered by penetrating or blunt trauma, instances of infection may occur without evident preceding injury. Predisposing factors encompass varicella, chronic skin conditions (such as decubitus or ischemic ulcers and psoriasis), and prior surgical procedures [37].

### 4.2. Streptococcal Toxic Shock Syndrome

Streptococcal toxic shock syndrome (STSS) is a rare exotoxin-induced complication of streptococcal pharyngitis. STSS often presents with an abrupt onset of high fever, hypotension, and multiorgan dysfunction. Patients may exhibit signs of shock, including altered mental status, tachycardia, and hypoperfusion of vital organs. Soft-tissue infections, such as cellulitis or necrotizing fasciitis, frequently precede STSS, serving as a portal of entry for the pathogen. Classically, the rash presents as a diffuse, blanching, macular erythroderma. Initially, it may manifest as a fleeting macular rash, primarily on the chest. The rash undergoes desquamation one to two weeks later, followed by complete peeling. Mucosal involvement may include a strawberry tongue and ulceration of the vaginal mucosa or conjunctival erythema. Patients may display disorientation or altered mental status without focal deficits [40]. Laboratory investigations in STSS often reveal hematological abnormalities, including leukocytosis, thrombocytopenia, and disseminated intravascular coagulation (DIC). These findings reflect the systemic inflammatory response and the potential for widespread microvascular thrombosis. Hypotension is present by definition, as are signs of multiple organ failure in at least two systems, including signs of acute renal or hepatic failure, signs of acute coagulopathy, and acute respiratory distress syndrome [41].

## 5. Diagnosis of GAS Infections

Diagnosis of GAS infections involves a comprehensive approach, including clinical evaluation, laboratory tests, and, in certain cases, imaging. The diagnosis of acute *Streptococcus pyogenes* infection is based on culturing bacteria from clinical specimens, while that of post-streptococcal disease is based on the detection of specific antibodies. Patients presenting with symptoms suggestive of GAS pharyngitis, without typical signs of viral infection, are eligible for microbiologic testing, including a rapid antigen detection test (RADT) or throat culture. Clinical features alone cannot reliably distinguish between GAS and viral pharyngitis, except in cases with viral symptoms of rhinorrhea, cough, or oral ulcers [27]. An RADT’s positive result obviates the need for throat culture due to the high specificity of the test. False-positive antigen results can be seen for patients previously diagnosed and/or treated for Group A *Streptococcus* or for patients colonized with non-pyogenes streptococcal species that carry the Lancefield group A antigen. The sensitivity of RADT for GAS is comparable to that of conventional throat culture. A negative RADT should be followed by a throat culture if a diagnosis of GAS infection cannot be ruled out. Although RADTs provide rapid results to allow early treatment decisions, culturing throat swabs for *Streptococcus pyogenes* remains the gold standard. Anti-streptococcal antibody titers are not recommended for the diagnosis of GAS pharyngitis because the test reflects previous infections. Following treatment, throat cultures or RADTs are not recommended routinely but may be considered in special circumstances. For the other acute infections, including those of the skin and the invasive ones, the culture of specimens is indicated in order to identify the bacteria in clinical samples. Imaging studies, such as radiography, ultrasound, and computed tomography (CT), may be performed to assess the extent of tissue involvement in cases of severe infections, such as necrotizing fasciitis. The diagnosis of post-streptococcal diseases is based on clinical history and findings compatible with the specific illness associated with the evidence of a preceding group A strep infection, including the isolation of GAS from the throat/skin or the detection of certain streptococcal antibodies (anti-streptolysin O and anti-DNase B). A four-fold increase in titer is considered the proof of antecedent streptococcal infection [27,29,33,42,43,44].

## 6. Treatment

The management of GAS pharyngitis remains disputed given the presence of multiple, varying guidelines. As a recent review highlights, these guidelines can be divided into three groups [43]. The first group recommends identifying GAS pharyngitis, treating it properly, and preventing possible sequelae. The second group considers GAS pharyngitis as a benign and self-limiting disease requiring treatment only in select cases. Finally, the third group, which includes guidelines from Australia and New Zealand, identifies two groups of patients at higher risk: patients of Maori or Aboriginal ethnicity, especially people living in rural areas, characterized by overcrowding and low socioeconomic status, and those with a previous history of acute rheumatic fever aged 3–40 years old. Once a diagnosis of GAS pharyngitis is confirmed, antimicrobial therapy should be promptly initiated to prevent both suppurative and non-suppurative complications, in particular, ARF, in which case the treatment should be initiated within 9 days of symptom onset [44,45].

Generally, given that GAS resistance to penicillin has not been detected to date, all guidelines agree on considering penicillin V as the drug of choice for the treatment of GAS pharyngitis [46]. Alternatively, if penicillin V is not available, Amoxicillin may be prescribed, also for better palatability [42]. Both Penicillin V and Amoxicillin are inexpensive, narrow-spectrum, and have a low rate of side effects. In cases with low compliance to these treatments, Penicillin G benzathine can be administered in a single intramuscular dose. For those patients who have a documented allergy to penicillins, a first-generation cephalosporin such as Cephalexin can be prescribed, particularly in non-anaphylactic forms of penicillin allergy, while clindamycin and azithromycin can be used in other cases [46]. It should be underlined that in recent years there has been an increase in the resistance of GAS to macrolides in Western countries [47]. Hence, if a macrolide is necessary, it is recommended to consider local resistance patterns and ensure bacterial susceptibility to this class of antibiotics [43]. Table 1 summarizes the antibiotic options for the treatment of GAS pharyngitis in children. Post-treatment throat culture to confirm the cure is not necessary unless symptoms persist for days following treatment [48]. Repeated positive tests for GAS without signs and symptoms of local inflammation are defined as a chronic GAS carrier state. These children are at low risk of immune-mediated complications and generally should not be treated [49]. Furthermore, they are unlikely to transmit the infection [50]. Eradication for the GAS carrier state must be taken into account in the following risk conditions: a local outbreak of GAS sequelae, including ARF, APSGN, or iGAS disease; an outbreak of GAS pharyngitis in a closed or semiclosed community; family or personal history of acute rheumatic fever; and multiple episodes of GAS pharyngitis occurring in a family for many weeks despite appropriate treatment [42,43,44,45,49]. Antibiotic options for eradication include clindamycin, penicillin, rifampin, and amoxicillin–clavulanic acid [46]. During the last few years, increased rates of invasive GAS infections in children have been reported in many countries [50,51]. Invasive GAS infections may be non-specific at the onset and may rapidly progress. Consequently, a high index of suspicion is essential to diagnose and treat the disease appropriately [20]. Antibiotic therapy remains the cornerstone of treatment in invasive infections, with the recommendation to initiate with a broad-spectrum antibiotic, subsequently modifiable once culture results are available. If GAS infection is confirmed, β-lactam antibiotics should be considered in susceptible cases. In case of penicillin allergy, macrolides are not recommended, and vancomycin is the preferred choice. The addition of an antitoxin antibiotic such as clindamycin or linezolid is recommended, especially in cases of necrotizing fasciitis and streptococcal toxic shock syndrome [20].

## 7. Antimicrobial Resistance

Even though GAS infections have historically responded well to β-lactam antibiotics, recent years have seen increasing reports of treatment failures in eradicating GAS from the throat in patients with GAS tonsillitis, with a rate of penicillin failure reported near 40% [52]. Studies have identified the gene encoding penicillin-binding protein 2X (pbp2x) in GAS strains with reduced β-lactam susceptibility; the lack of diffusion of this particular PBP2x polymorphism is probably related to the small evolutionary advantage conferred by this variant [53].

Multiple hypotheses exist regarding the possible mechanisms of GAS penicillin reduced susceptibility. According to one, the internalization of GAS in epithelial cells effectively makes eradication more difficult due to the poor penetration of penicillins into the tonsillar tissues. A second hypothesis posits a synergistic relationship between GAS and bacteria colonizing the oral cavity (e.g., *Moraxella catarrhalis*), facilitating the adhesion and persistence of GAS, or, alternatively, protection induced by other β-lactamase-producing commensal bacterial species such as *Staphylococcus aureus* [52,54,55]. Since 1990, there has been a notable increase in macrolide resistance among GAS strains, with resistance rates varying widely across geographic regions. Some European countries report resistance rates below 4%, while rates exceed 40% in some Asian countries. Mechanisms of resistance include target site modification or drug efflux, particularly affecting erythromycin. Fortunately, a decrease in resistance rates has been described in some countries under antimicrobial stewardship programs [47,56,57]. The prevalence of resistance to fluoroquinolones ranges from infrequent occurrences at low levels to sporadic instances at high levels. Conversely, resistance to tetracycline is characterized by its low occurrence and is typically associated with mutations in genes encoding efflux pumps, which are relatively rare in streptococci. Notably, the genes of macrolide resistance are collocated on mobile elements near those responsible for tetracycline resistance.

## 8. The Current Status of GAS Vaccines

A GAS vaccine is not yet available, but the urgency of developing one is supported by the fact that GAS still ranks among the top ten infectious diseases causing mortality worldwide and remains one of the most frequent pathogens responsible for upper respiratory tract infections (pharyngitis and tonsillitis). The GAS global disease burden may be underestimated given the lack of epidemiological data from underdeveloped countries [58]. A GAS vaccine should be affordable at all income levels and should be allocated across all countries, regardless of their economic status. A worldwide distribution could prevent many cases of superficial diseases and deaths, reduce throat colonization, and decrease infections transmitted from asymptomatic carriers. Indirect benefits would include a reduction in antibiotic use and consequently a mitigation of antimicrobial resistance.

Several obstacles hinder the development of a GAS vaccine. Firstly, healthcare interventions in high-income countries, focusing on the prompt diagnosis and early treatment of sore throats, have reduced mortality and complications related to GAS infections, thus diminishing the demand for vaccines in these regions. Secondly, the shortage of funding for the development of a vaccine is consequent to the disproportionate need in low-income countries, where there is often insufficient return on investment. Certainly, the vast majority of cases of ARF, RHD, APSGN, and iGAS occur in developing countries, which are characterized by health inequities, poverty, and social disadvantage, suggesting that social determinants of health (SDHs) are a major driver of GAS infection persistence [59,60]. The GAS distribution is very different between developed countries, where circulating strains are few, and low-income settings, where the rate of circulating strains is higher. Social and environmental factors (such as overcrowding or comorbidities) contribute to bacterial growth and transmission, resulting in greater genetic diversity. This differential distribution, in addition to the extensive *emm*-type diversity (more than 250 types of *Streptococcus pyogenes*), is a great challenge for the development of a GAS vaccine, which should be able to cover multiple serotypes, leading to cross-serotype protection [8]. Another impediment to progress is the paucity of suitable animal models susceptible to GAS infection—indeed, mice do not have tonsils, while non-human primates are expensive and complex to use, requiring specialized facilities and personnel. Moreover, a delay in development is related to the fact that trials should be conducted first in healthy adults and after many years reach those phases in which the real target vaccine population is tested (specifically children and adolescents who are subject to the major burden of GAS infection) [61]. Another obstacle to vaccine development is the limited knowledge of the molecular mechanisms associated with complications caused by Group A *Streptococcus* infection. When developing a GAS vaccine, it is necessary to avoid the risk of autoimmune complications due to passive immunization. One of the reasons behind the discontinuation of trials is the onset of rheumatic fever in some patients immunized with a candidate vaccine. Since the beginning of the 20th century, many studies about vaccines able to prevent scarlet fever were conducted, leading to the first trial in 1923. Between 1923 and the late 1960s, different vaccine products were tested. In 1968, a candidate vaccine based on the M protein caused acute rheumatic fever in at least two children enrolled in a trial; this event led to the discontinuation of trials for decades. In December 2005, the regulation was revoked by the Food and Drug Administration (FDA), and since 2006, many initiatives for vaccine research have been supported by the World Health Organization (WHO) to ensure the development of a safe, effective, and affordable GAS vaccine [60].

Two types of GAS vaccine are available: those focused on the major cell surface protein (M-protein-based vaccines) and those targeting different GAS factors, including toxins, proteins, and capsule constituents (non-M-protein-based vaccines) (see Table 2 and Table 3) [58,62].

Currently, among the M-protein-based vaccines, three candidates have completed or begun a phase 1 clinical trial. Specifically, Streptanova, a 30-valent vaccine, has completed a phase 1 clinical trial and was found to be effective against 72 GAS *emm*-types; StreptIncor, a polypeptide vaccine with B and T cell epitopes, has begun a phase 1 trial and seems to prevent infection against M1, M5, M12, M22, and M87 GAS strains; MJ8Vax is still in the preclinical research stage, and, if successful, it will be the first nasally administered GAS vaccine. The 26-valent M-protein-based vaccine completed a phase 2 trial, being well-tolerated and immunogenic in healthy adults [62,63]. The non-M-protein-based vaccines include many candidates in the preclinical stage: carbohydrate vaccines (GAC), Combo4 and Combo5, TeeVax, Vax-A1, 5CP, Spy7, and SPy-2191. The Combo vaccine is a multiple antigen composed of SLO (a pore-forming toxin), SpyAD (a surface-exposed adhesin), SpyCEP (a protease), and Group A carbohydrate (GAC) and should soon enter clinical development [62]. At present, considering the ongoing development of vaccine candidates, the prevention strategies for GAS infections include early diagnosis and treatment of GAS tonsillitis, screening of asymptomatic cases in hospital and care facilities, post-exposure prophylaxis of vulnerable groups, improved hand hygiene practices, improvements in the quality of housing, and reductions in overcrowding. Lastly, improved surveillance and epidemiological investigation should be emphasized, especially in low-resource settings, as essential practices in the prevention and control of the spread of GAS diseases [64]. Many authors have reported that a cost-effective *Streptococcus pyogenes* vaccine could lower the morbidity and mortality burden in all income settings [65]. A static cohort model has been developed to estimate the projected health impact of GAS vaccination using country-specific demographic data (high- and low-income countries). In this model, available online, the vaccination impact is estimated in terms of reduction in the burden of several major GAS disease states (both throat and skin infections, but also post-streptococcal complications), sequelae, and deaths due to severe GAS diseases. The app shows the predicted lifetime health benefits if a GAS vaccine were to be distributed, underlining the direct reduction in disease burden on the bases of vaccine efficacy, coverage, and vaccine-derived immunity [66].

## 9. Conclusions and Future Directions

Although GAS is responsible for trivial infections such as pharyngitis, it is increasingly evident that the suppurative and non-suppurative complications should not be underestimated given that this pathogen is one of the most significant causes of global morbidity and mortality. In the last few years, a progressive increase in *emm*-type numbers has been identified along with the emergence of the first forms exhibiting reduced susceptibility to penicillins. There is therefore an urgent need for vaccine development against Group A *Streptococcus*, especially in low-resource settings where the diagnosis and treatment of pharyngitis is not always feasible and where the complications of GAS infections occur most frequently. Continued monitoring of the incidence of GAS infections, especially in low-resource settings, is important in order to assess the impact of preventive measures and the effectiveness of future vaccination strategies.

## Figures and Tables

**Table 1:** Suggested antibiotic treatments for GAS acute pharyngitis in children.

Antibiotic	Dose	Duration	Considerations
Penicillin V (oral)	≤27 kg of body weight: 250 mg 2–3 times a day >27 kg: 500 mg 2–3 times a day	10 days	Preferred treatment
Amoxicillin (oral)	50 mg/kg once a day (max dose: 1 g)	10 days (in low-risk patients, a 5-day course of treatment should be considered)	Preferred treatment
Penicillin G benzathine (intramuscular)	≤27 kg of body weight: 600,000 U >27 kg: 1,200,000 U	1 dose	To be considered in case of poor treatment adherence
Cephalexin	40 mg/kg/day 2 times a day (max dose: 500 mg)	10 days	In children with non-anaphylactic penicillin allergy
Azithromycin	12 mg/kg on day 1 and 6 mg/kg on days 2–5 (max dose: 500 mg)	5 days	In children with anaphylactic penicillin allergy; consider macrolide resistance
Clindamycin	20 mg/kg/day 3 times a day (max dose: 300 mg)	10 days	In children with anaphylactic penicillin allergy; consider clindamycin resistance

**Table 2:** GAS vaccines and their stages of development, M-protein-based vaccines.

Vaccine Name	Target Antigen	Stage of Development	Characteristics
M-Protein 26-valent vaccine	M-Protein	Phase II	Effective against 26 different serotypes of GAS; safe and effective in 26 healthy adult volunteers; no occurrence of cross reaction with human tissue
M-Protein 30-valent vaccine (Streptanova)	M-Protein	Phase I	Four recombinant proteins; covers 30 GAS serotypes; safe in healthy adults without causing autoimmunity
M-Protein C repente epitope (StreptInCor)	M-Protein	Phase I	A 55-amino acid peptide from the C-terminal region of the M-protein (highly conserved among GAS serotypes). Animal immunization studies have shown high levels of specific antibodies with no cross-reactivity to cardiac proteins
MJ8Vax	M-Protein	Phase I	J8 is the smallest epitope in the C region of the M protein binding to CRM (an inactive and non-toxic form of DT); it is conjugated with K4S2-CRM. It will be the first nasally administered GAS vaccine
PMA-P-J8	M-Protein	Preclinical	J8 B-cell epitope of M protein, PADRE, and PMA expression of IgG and mucosal IgA after a single immunization
P*17/K4S2	M-Protein	Preclinical	Derivative of the peptide in the C region of the M protein. In mice a single immunization resultes protective against skin and invasive disease.
BP-p*17-S2	M-Protein	Preclinical	Sintetized using biopolymer particles. In mice model led to significant reduction in GAS load. Low cost vaccine.

PADRE: pan HLA-DR-binding epitope; PMA: poly methyl acetate.

**Table 3:** GAS vaccines and their stages of development, non-M-protein-based vaccines.

	Target Antigen	Stage of Development
GAC	GAC	Preclinical
Combo4	SpyCEP, SLO SpyAD, GAC	Preclinical
Combo5	SLO, SpyCEP, SCPA, ADI, TF	Preclinical
TeeVax	T antigen	Preclinical
VAX-A1	GACprSpyAD, SLO, SPCA	Preclinical
SCP	SrtA, SCPA, SpyAD, SpyCEP, SLO	Preclinical
Spy7	SCAPA, OppA, PulA, SpyAD, Apy1228, Spy1037, Apy0843	Preclinical
Spy_2191	Spy_2191	Preclinical

ADI: argininedeiminase; SCPA: Streptococcus C5a peptidase; TF: trigger factor; GAC: group A carbohydrate; SLO: streptolysin O.

## Data Availability

Not applicable.
